# Applications of MOF-Based Nanocomposites in Heat Exchangers: Innovations, Challenges, and Future Directions

**DOI:** 10.3390/nano15030205

**Published:** 2025-01-27

**Authors:** Talha Bin Nadeem, Muhammad Imran, Emad Tandis

**Affiliations:** 1 Department of Mechatronics and Biomedical Engineering, College of Engineering and Physical Sciences, Aston University, Birmingham B4 7ET, UK or talhanadeem@neduet.edu.pk (T.B.N.); e.tandis@aston.ac.uk (E.T.); 2 Department of Mechanical Engineering, NED University of Engineering and Technology, Karachi 75270, Pakistan; 3 Energy Systems Group, Energy and Bioproduct Research Institute, College of Engineering and Physical Sciences, Aston University, Birmingham B4 7ET, UK

**Keywords:** metal–organic frameworks, MOFs, heat exchanger, nanocomposites, thermal conductivity, energy efficiency, heat transfer enhancement, fouling resistance

## Abstract

Metal–organic frameworks (MOFs) have garnered significant attention in recent years for their potential to revolutionize heat exchanger performance, thanks to their high surface area, tunable porosity, and exceptional adsorption capabilities. This review focuses on the integration of MOFs into heat exchangers to enhance heat transfer efficiency, improve moisture management, and reduce energy consumption in Heating, Ventilation and Air Conditioning (HVAC) and related systems. Recent studies demonstrate that MOF-based coatings can outperform traditional materials like silica gel, achieving superior water adsorption and desorption rates, which is crucial for applications in air conditioning and dehumidification. Innovations in synthesis techniques, such as microwave-assisted and surface functionalization methods, have enabled more cost-effective and scalable production of MOFs, while also enhancing their thermal stability and mechanical strength. However, challenges related to the high costs of MOF synthesis, stability under industrial conditions, and large-scale integration remain significant barriers. Future developments in hybrid nanocomposites and collaborative efforts between academia and industry will be key to advancing the practical adoption of MOFs in heat exchanger technologies. This review aims to provide a comprehensive understanding of current advancements, challenges, and opportunities, with the goal of guiding future research toward more sustainable and efficient thermal management solutions.

## 1. Introduction

**Figure 1:**
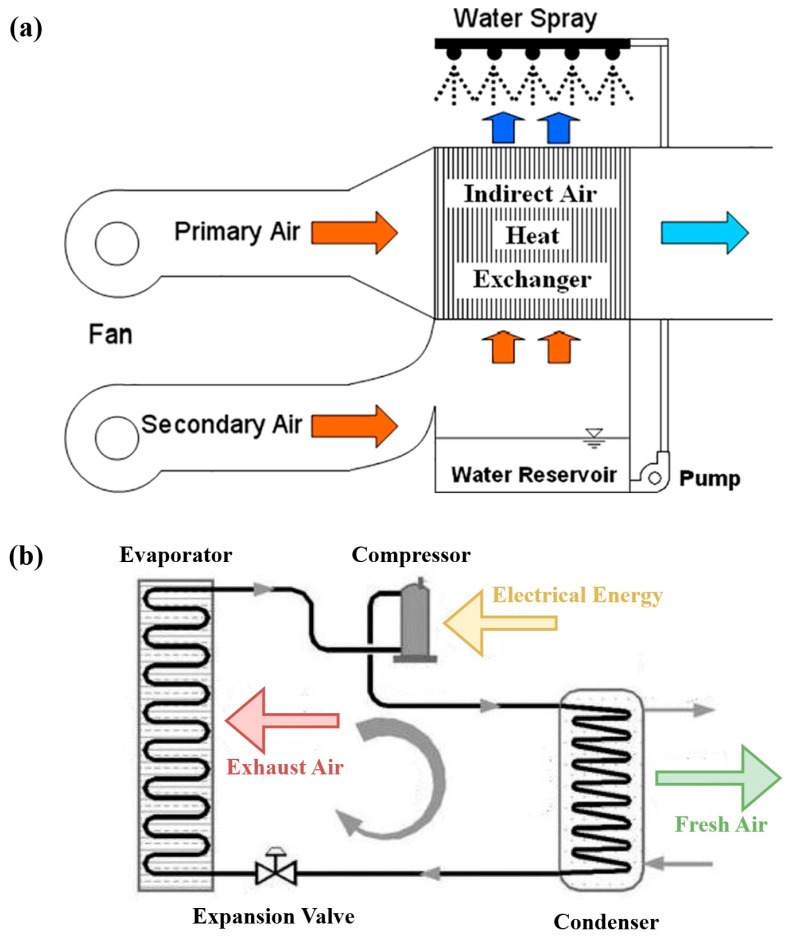
Schematics of (**a**) indirect evaporative cooling system [7] and (**b**) heat pumps [8], illustrating their working principles (reproduced with permission from Wiley and Elsevier).

Heat exchangers are essential components across numerous engineering applications, including HVAC systems, automotive designs, and industrial manufacturing. These systems demand materials that can effectively conduct heat at high rates while maintaining stability and minimal thermal expansion across a range of operating temperatures [1]. In applications such as air conditioning, refrigeration, and energy recovery, heat exchangers are critical to optimizing system efficiency, cost, and compactness. Similarly, heat exchangers play a pivotal role in both cooling and heating applications, acting as the core component for energy transfer between fluids in these systems. Similarly, in indirect evaporative cooling, heat exchangers enable the transfer of heat from the warm air inside a building to the cooler exhaust air outside, facilitating temperature reduction without adding moisture to the air [2]. Figure 1a represents the schematic diagram of an indirect evaporative cooling system, depicting its working principle. The efficiency of this process heavily depends on the heat exchanger’s design, as it determines the thermal performance and overall energy efficiency of the system. A well-designed heat exchanger maximizes the surface area for heat transfer while minimizing the energy required to drive the process, thereby improving the cooling performance and reducing operational costs. However, the design must be optimized to balance thermal conductivity, airflow, and the resistance to fouling, which can significantly impact system efficiency over time [3]. Consequently, in heat pumps, heat exchangers are crucial for transferring heat between the refrigerant and the environment. In heating, heat exchangers extract heat from the outside air or ground and release it inside [4]. The effectiveness of heat exchangers in heat pumps directly influences the system’s coefficient of performance (COP), which is a measure of efficiency. A higher COP translates to better energy utilization, which is particularly important in residential and commercial applications where energy efficiency and cost savings are prioritized [5]. In both applications, selecting the right type of heat exchanger—whether air-to-air, air-to-water, or ground-coupled—is crucial for optimizing the system’s performance [6]. Figure 1b represents the schematic diagram of a heat pump, depicting its working principle.

**Figure 2:**
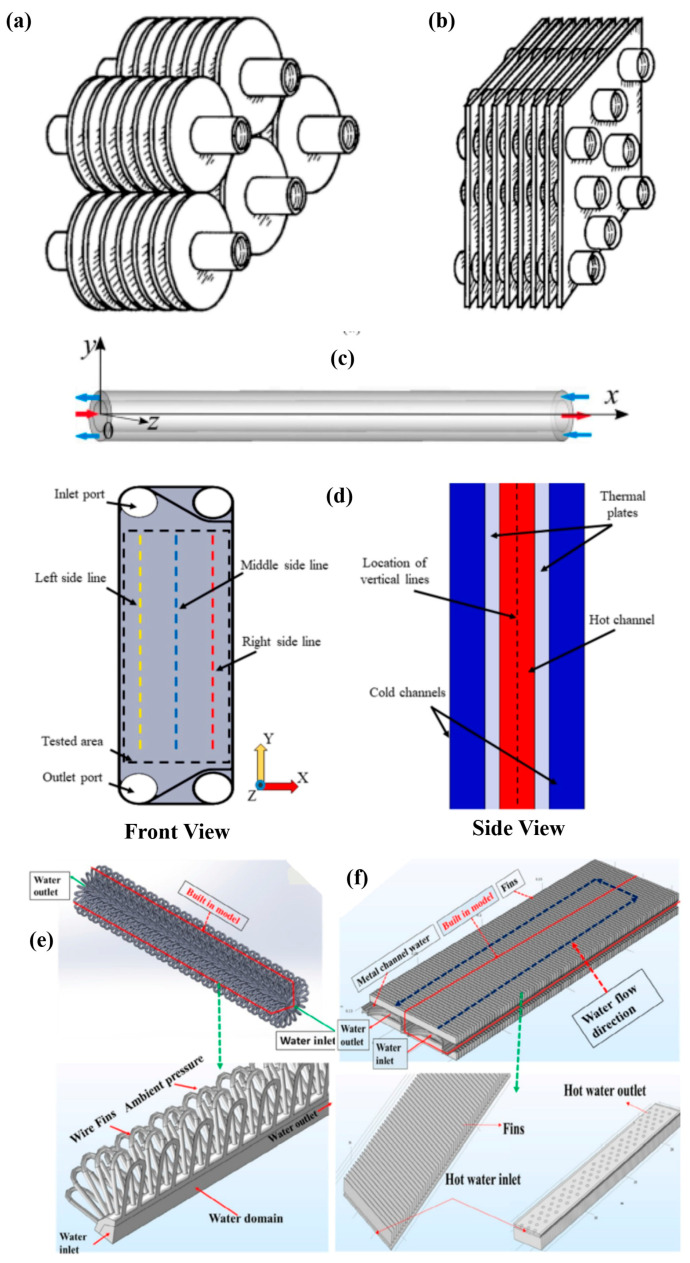
Schematics of (**a**) individual fin [10], (**b**) continuous fins [10] in tube finned, (**c**) double pipe [11], (**d**) plate type [12], (**e**) wire-finned [13], and (**f**) micro-channel heat exchanger [13] commonly used in HVAC applications (reprinted with permission from Wiley and Elsevier).

Traditional designs, like fin-and-tube or plate-fin heat exchangers made from materials such as copper, aluminum, or steel, are widely used. Figure 2 provides illustrations of widely used heat exchangers in HVAC. Numerous studies focus on improving heat transfer in these conventional systems. However, the performance limitations of metal-based heat exchangers in certain applications have driven interest in exploring alternative designs with different materials [9]. Despite their widespread use, several efficiency bottlenecks limit the performance, longevity, and reliability of these devices.

Key issues include thermal resistance, fouling, and material degradation, each of which affects the heat exchanger’s efficiency, operational cost, and maintenance needs [14]. Thermal resistance, a key limitation in heat exchangers, hinders efficient heat transfer between mediums. It is influenced by the heat exchanger’s design, material thermal conductivity, and surface thermal boundary layers, directly affecting overall system performance [15]. In metallic heat exchangers, materials like copper and aluminum are used due to their high thermal conductivity. However, limitations arise, as these metals exhibit high thermal expansion and can degrade over time under harsh conditions, leading to increased thermal resistance [16]. Advanced materials, such as MOFs, have shown promise in enhancing thermal conductivity while maintaining structural stability. Researchers have also explored nanocomposites to improve the heat transfer rate, especially in systems where high-temperature stability is critical [17]. Fouling, caused by the buildup of materials like dust, biofilms, or mineral deposits on heat exchanger surfaces, adds thermal resistance and reduces heat transfer efficiency. This can decrease heat transfer rates by 30–50%, significantly increasing energy consumption to maintain desired performance [18,19]. Fouling is often more prevalent in heat exchangers used in environments with high concentrations of particles or biological matter, such as wastewater treatment facilities and cooling towers [20]. The cost of dealing with fouling, through either frequent maintenance or more complex cleaning processes, significantly adds to the operational cost of heat exchanger systems [21]. Antifouling coatings and self-cleaning mechanisms are emerging as potential solutions to mitigate these effects. For example, nanocomposite coatings that combine MOFs with hydrophobic materials can minimize fouling while maintaining high thermal conductivity [22]. Biofouling involves microbial growth forming biofilms on surfaces. Biofouling can obstruct flow channels, decrease membrane permeability, and necessitate frequent cleaning, thereby elevating maintenance costs and downtime [23,24]. Mechanical failure in water treatment systems can result from material degradation, corrosion, or stress-induced damage. Factors such as biofouling exacerbate these issues by creating differential pressures and localized corrosion sites, leading to equipment malfunction or failure [25]. For instance, biofouling-induced corrosion can compromise the structural integrity of pipelines and membranes, resulting in leaks or ruptures [26]. Fouling layers in heat exchangers decrease thermal conductivity, reducing energy efficiency. Hybrid coatings and periodic cleaning methods have been implemented to counteract fouling, ensuring consistent heat transfer performance. Antibacterial treatments also prevent biofilm-induced corrosion, extending equipment life [27]. Biofouling in water-related processes, such as reverse osmosis membranes and pipelines, reduces water flow and increases energy consumption. Antimicrobial coatings, such as the hybrid polyaniline (PANI)-halamine introduced by Weiss et al. [28], effectively prevent biofilm formation, improving system reliability and minimizing downtime.

**Figure 3:**
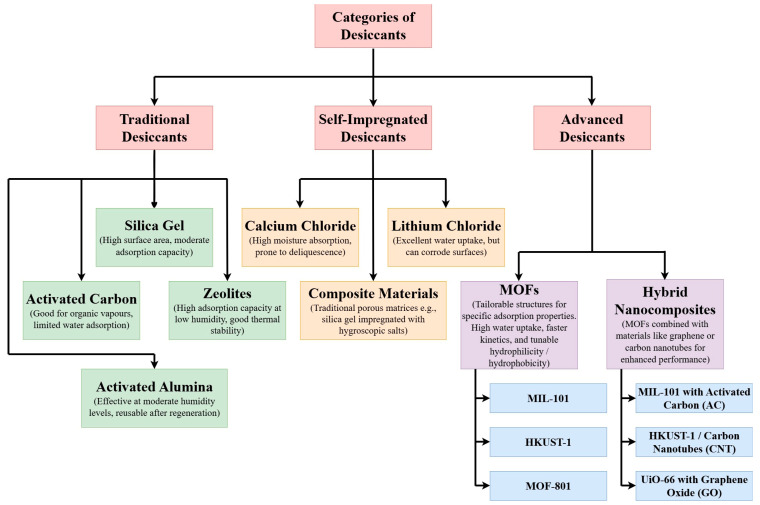
Categorization of desiccant materials that can be used as coating material in heat exchangers.

Material degradation is another major bottleneck in heat exchanger performance, particularly in systems exposed to corrosive or high-temperature environments. Common materials like aluminum, copper, and steel are prone to corrosion, which can reduce their effectiveness and shorten the lifespan of the heat exchanger. Corrosion not only lowers thermal conductivity but also makes the material more susceptible to mechanical failure [29]. In high-temperature applications, such as those found in power generation or chemical processing, degradation can also occur due to thermal cycling, which leads to metal fatigue and cracking [30]. Researchers are exploring MOF-based composites and other advanced materials to combat these issues by improving thermal stability and reducing degradation rates [31]. For instance, integrating MOFs with carbon-based materials like graphene can offer a combination of high thermal conductivity and resistance to thermal and mechanical stress, making them more durable than conventional materials in challenging environments [32,33]. Advancing heat exchanger technology requires addressing challenges like thermal resistance, fouling, and material degradation. MOFs and MOF-based nanocomposites, with their high surface area and customizable properties, show potential in enhancing heat transfer efficiency, resisting corrosion, and reducing fouling for improved performance [34,35]. For example, in one study, a MOF composite integrated with graphene achieved a 25% reduction in thermal resistance and a 40% improvement in fouling resistance compared to conventional metal-based heat exchangers [34]. Such innovations highlight the potential of MOF-based materials to overcome long-standing challenges in heat exchanger design. Figure 3 illustrates the categorization of desiccant materials that can be used as coating material in heat exchangers.

**Figure 4:**
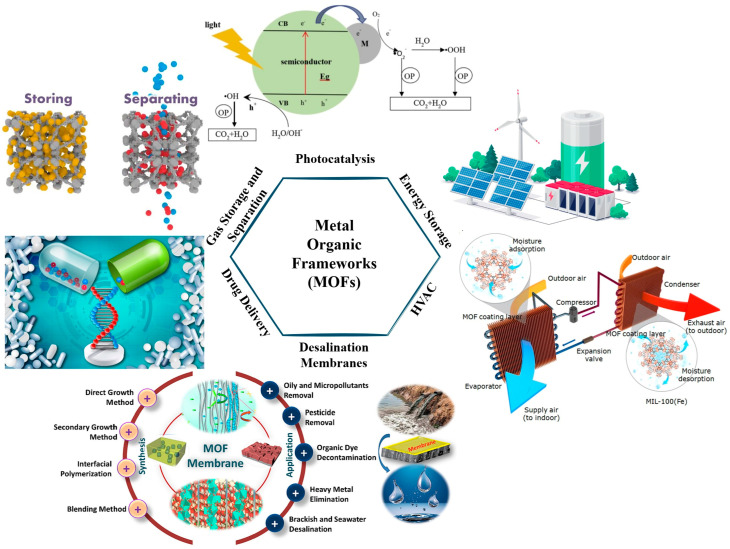
Applications of metal–organic frameworks (MOFs) in different research areas.

MOFs are highly porous materials consisting of metal ions coordinated to organic ligands, forming intricate network structures with exceptionally high surface areas and tunable properties. Due to these characteristics, MOFs have gained attention in applications such as gas storage, catalysis, and thermal management. However, by combining MOFs with other materials—such as metals, polymers, or carbon-based substances—researchers have created MOF-based nanocomposites that can further enhance thermal properties, making them especially promising for applications in heat exchangers and other energy-intensive systems [36]. MOFs are known for their vast internal surface area, often exceeding that of traditional porous materials, which enables greater interaction with heat transfer media. This high surface area plays a key role in MOF-based nanocomposites’ ability to improve thermal management in heat exchangers [37]. For instance, studies have demonstrated that MOFs with large surface areas can effectively adsorb and release thermal energy, making them suitable for thermal energy storage and transfer. Additionally, the adjustable pore size in MOFs allows them to accommodate various guest molecules and even modify the thermal flow rate by controlling gas or fluid adsorption [38]. Pure MOFs often have lower thermal conductivity than metals, which limits their direct use in heat exchangers. However, by integrating MOFs with conductive fillers such as graphene oxide (GO), carbon nanotubes (CNTs), or metallic particles, researchers can significantly enhance their thermal conductivity, achieving composite materials that efficiently manage heat [39]. Graphene-based MOF composites, for example, leverage the thermal conductivity of graphene while retaining the high porosity and adsorption properties of the MOF framework, creating materials suitable for high-performance thermal applications. This approach addresses one of the primary limitations of traditional MOFs, making them more viable for practical applications in thermal management systems [40]. Figure 4 represents some of the research areas where MOFs have been used in recent years.

MOFs have gained attention for their versatility across various applications beyond heat exchangers such as water treatment and gas separation. In water treatment, MOFs demonstrate exceptional performance in removing heavy metals, organic pollutants, and dyes due to their high surface area and customizable pore structure [41]. For instance, Zr-based MOFs like UiO-66 effectively adsorb arsenic and lead ions from contaminated water [42] due to their chemical stability, high surface area, and abundant functional groups such as hydroxyl (-OH) and carboxyl (-COOH) on their frameworks. These functional groups interact strongly with toxic metal ions through coordination bonds or electrostatic attraction, facilitating their removal from contaminated water. Additionally, UiO-66’s tunable pore size allows for efficient diffusion and trapping of these ions, making it an excellent candidate for water purification applications [43]. Similarly, MOFs are highly effective in gas separation, where their tunable pore sizes enable selective adsorption of gases like CO_2_, CH_4_, and H_2_. ZIFs are particularly notable for their efficiency in CO_2_ capture and storage [44]. Their imidazolate linkers create a hydrophobic environment, enhancing CO_2_ selectivity while minimizing interference from water vapor, making them ideal for carbon capture applications [45]. Furthermore, MOFs are being explored in drug delivery, catalysis, and energy storage due to their structural flexibility and functional diversity. Addressing these broader applications highlights the multifunctionality of MOFs and underscores their potential for innovative solutions across diverse fields.

A major advantage of MOF-based nanocomposites is their chemical and structural tunability, which allows for customized thermal properties. MOFs can be synthesized with a wide range of metal nodes and organic linkers, providing flexibility in terms of pore size, surface chemistry, and thermal response [46]. Furthermore, when MOFs are combined with polymers, the resulting composites gain mechanical stability and can be engineered to resist degradation under thermal cycling. For instance, incorporating polymers such as polydimethylsiloxane (PDMS) with MOFs can produce materials that maintain structural integrity at elevated temperatures, extending the lifespan of heat exchangers in high-temperature environments [47]. Another significant advantage of MOF-based nanocomposites lies in their excellent adsorption capacity, which enhances their thermal management capabilities [48,49]. MOFs have been shown to adsorb various gases efficiently, making them ideal candidates for applications in adsorption-based cooling systems. By incorporating MOFs with metals or metal oxides, the adsorption–desorption properties of these composites can be optimized for rapid thermal response, a crucial feature for efficient heat exchangers [50]. This adaptability not only improves energy efficiency but also contributes to reducing the overall operational costs of cooling systems [51]. In addition to their thermal properties, MOF-based nanocomposites exhibit improved resistance to fouling and corrosion, common challenges in heat exchangers. For example, by integrating hydrophobic MOFs with anti-corrosive metals, researchers have developed nanocomposites that can minimize fouling from organic and inorganic deposits, thereby preserving heat transfer efficiency over time. This feature is especially beneficial in applications where maintenance and cleaning are difficult or costly [52].

The development of advanced materials for efficient thermal management is a critical focus in engineering, especially in the design of heat exchangers, which are central to systems such as HVAC, energy storage, and industrial processes. This review paper aims to examine the growing body of research on MOF-based nanocomposites as promising materials in heat exchangers. Recent advancements indicate that these composites, known for their high surface area, tunable thermal properties, and resilience in extreme environments, could significantly enhance heat exchanger efficiency. However, while MOF-based nanocomposites show potential, their practical application is challenged by factors such as thermal stability, material degradation, and scaling limitations. This review will explore these innovations and examine technical challenges to highlight the current state of MOF-based nanocomposites in heat exchanger applications. By addressing the gaps in existing research, this paper seeks to provide a comprehensive overview of the field and propose future research directions, including potential material modifications, synthesis techniques, and structural optimizations. This review is intended to serve as a resource for researchers and engineers, aiming to advance the practical applications of MOF-based nanocomposites in energy-efficient, sustainable heat exchanger designs.

The growing demand for energy-efficient thermal management systems, particularly in industries reliant on heat exchange processes, has driven interest in innovative materials like MOFs due to their exceptional thermal properties and tunable functionalities. However, despite significant advancements in the synthesis and application of MOF-based materials, there remains a fragmented understanding of their performance and practical integration into industrial heat exchangers. This review provides a comprehensive examination of the current state of MOFs in enhancing heat exchanger efficiency, addressing not only their potential but also the challenges in scaling these materials for real-world use. Section 2 delves into the impact of MOFs on heat exchanger performance, examining their role in improving heat transfer and reducing fouling. Section 3 explores recent innovations and ongoing challenges in integrating MOF-based materials into heat exchangers, highlighting advancements in synthesis techniques, surface functionalization, and industrial feasibility. Finally, Section 4 identifies key research gaps and outlines future directions for the development of MOF-based nanocomposites, aiming to guide future research and accelerate the adoption of MOF technologies in energy-efficient applications. By addressing these critical areas, this review aims to bridge the gap between academic research and practical implementation, supporting the development of more sustainable and effective heat exchange solutions.

## 2. MOF-Based Nanocomposites for Heat Exchanger Performance

### 2.1. High-Performance MOF Nanocomposites for Thermal Conductivity

Incorporating MOFs into metals and polymers enhances thermal conductivity in heat exchangers by improving thermal transport pathways and reducing interfacial thermal resistance, often termed as Kapitza resistance, which arises at interfaces between materials with differing thermal properties, particularly at the nanoscale [53]. This phenomenon arises due to the mismatch in acoustic phonon properties—vibrational energy carriers—of the materials on either side of the interface [54]. When heat flows across the interface, phonons encounter impedance due to differences in lattice structure, phonon density of states, or material stiffness. This impedance reduces the efficiency of thermal energy transfer, creating a bottleneck for heat conduction [55]. Kapitza resistance is particularly significant in systems involving nanoscale materials, such as nanocomposites or interfaces in electronic devices, where the surface-to-volume ratio is high [56]. The effect of Kapitza resistance is a reduction in overall heat transfer efficiency, which can limit the performance of thermal management systems. For example, in electronic devices, high Kapitza resistance at material interfaces can lead to localized overheating, affecting reliability and longevity. Minimizing Kapitza resistance through techniques such as interface engineering, use of intermediate materials to bridge phonon mismatch, or applying thermal interface materials can improve heat transfer efficiency across interfaces [57]. By embedding MOFs, which possess a highly porous structure and customizable surface chemistry, researchers can create a network of micro- and nanopores that facilitates smoother energy transfer across interfaces [58,59]. By filling gaps at the interface, MOFs create a more uniform thermal contact area, which decreases Kapitza resistance. The smooth energy transfer results from the mitigation of phonon scattering caused by mismatches in acoustic properties between materials [60]. The ordered structure of MOFs ensures that heat conduction is not only through direct contact but also facilitated by the framework’s intrinsic thermal conductivity. This supports efficient energy propagation.

The enhancement in thermal conductivity from MOFs is due to improved phonon transport, as MOFs create ordered pathways that reduce scattering events at material interfaces. This effect is particularly beneficial in polymer composites, aligning thermal pathways and promoting efficient heat conduction [61]. For example, incorporating MOFs with high thermal conductivity into polymers has been shown to enhance their thermal conductivity, making these composites suitable for high-performance thermal applications [62]. Thermal conductivity remains a critical factor in emerging technologies and applications, including heat exchangers, aerospace, and mechanical instrumentation. Many advanced smart materials used in electronic devices generate heat during operation, leading to temperature increases that can degrade their performance. Elevated temperatures may also compromise the mechanical stability and functionality of polymeric components. To address this challenge, various strategies have been proposed to improve the thermal conductivity of polymers, with most focusing on the integration of thermally conductive nanoparticles [63].

**Figure 5:**
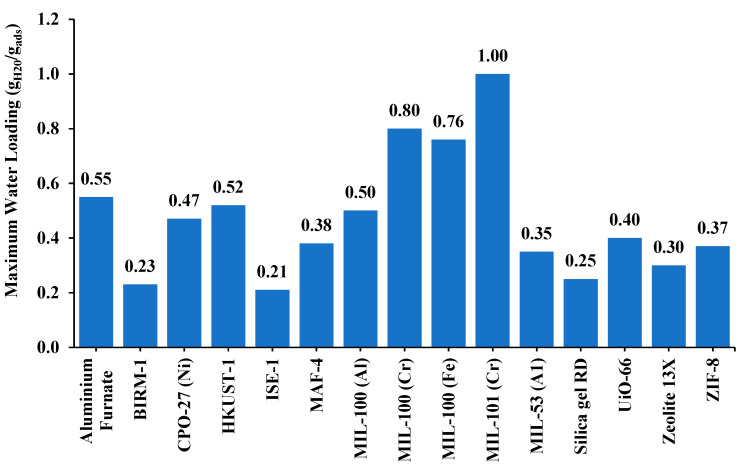
Maximum water vapor uptake (g_H20_/g_abs_) of various MOFs used in heat pumps.

Certain MOFs, such as MIL-101, UiO-66, and HKUST-1, have shown remarkable promise in enhancing thermal conductivity when incorporated into heat exchanger materials. MIL-101, with its large pore structure and high BET surface area of 3873 m^2^/g, facilitates efficient heat transfer and has been widely studied for improving thermal transport in composite systems [64,65]. MIL-101 has thermal conductivity ranging from 0.83 to 0.86 W/m-K. UiO-66, known for its exceptional stability and tunability, enables strong thermal conductivity enhancements, particularly in polymer composites [66,67]. UiO-66 has a BET surface area of 1041 m^2^/g. HKUST-1, composed of copper nodes and organic linkers, also demonstrates superior thermal properties, helping reduce interfacial thermal resistance in metal composites [68,69]. These MOFs contribute significantly to optimizing heat exchanger efficiency through enhanced thermal pathways. Several MOFs are used in adsorption heat pumps, and they have different water uptake. Some of these can be seen in Figure 5, for which the original data values have been extracted from [70,71,72,73,74,75,76,77,78,79,80,81,82].

**Figure 6:**
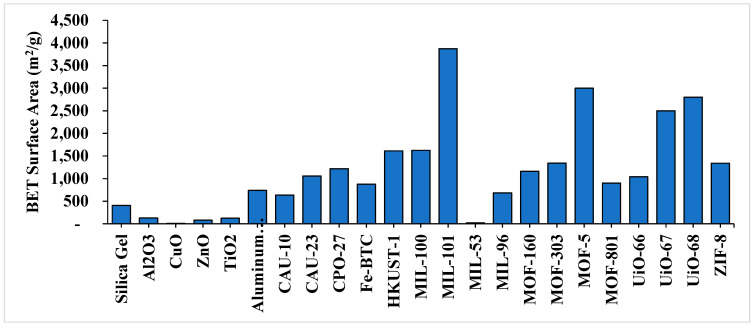
A schematic representation of the ultrahigh porosity of MOFs in heat transfer systems, highlighting their surface area in comparison with commonly used silica gel and commonly used metal oxides.

Thermal conductivity is a critical property for materials used in heat transfer applications. The experimental measurement of MOF conductivity is highly influenced by pore size. A summary of some results on the thermal conductivity of MOFs is presented in Table 1. Similarly, Figure 6 illustrates that the Brunauer–Emmett–Teller (BET) surface area of MOFs significantly surpass those typically found in conventional porous materials like silica gel and some widely used metal oxides. The original data have been gathered from [83,84,85,86,87,88,89,90,91,92,93,94,95,96,97].

**Figure 7:**
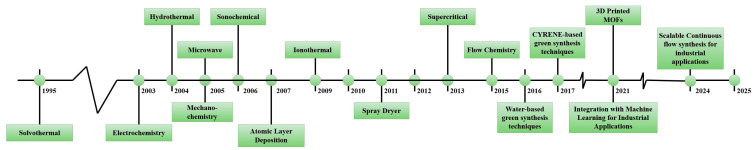
The development of MOF synthesis method over the years (updated and reproduced with permission from the Royal Society of Chemistry).

Embedding MOFs within metal and polymer matrices is an effective strategy to leverage the thermal properties, mechanical strength, and structural tunability of MOFs. These composites are especially valuable in heat exchanger applications, where both thermal conductivity and mechanical stability are critical. This section reviews key methods for embedding MOFs into metal and polymer matrices, highlighting techniques like in situ growth, physical blending, and surface functionalization. Figure 7 illustrates the MOF synthesis method over the years [111].

In situ growth is a widely used approach for embedding MOFs in both metal and polymer matrices. In this method, MOF crystals form directly within the matrix material, resulting in a composite with strong interfacial adhesion and uniform distribution. For metal matrices, in situ growth involves immersing the metal substrate in a solution containing MOF precursors, allowing MOF crystals to nucleate and grow on the metal surface. This process promotes thermal conductivity, as it creates continuous thermal pathways between the MOF and metal, reducing thermal boundary resistance [112]. In polymer matrices, in situ growth can involve dissolving MOF precursors within a polymer solution, which leads to MOF crystallization as the polymer matrix solidifies. This method ensures a well-integrated composite structure with enhanced mechanical properties and homogeneity [113].

Physical blending involves mixing pre-synthesized MOF particles with metal or polymer matrices, often through mechanical blending or extrusion. While cost-effective and scalable, this method faces challenges in achieving uniform dispersion and strong interfacial bonding, which can limit thermal transport enhancement [114]. In polymer matrices, physical blending typically involves dispersing MOF particles in a polymer solution or melt, followed by casting or extrusion to form a composite. Advanced mixing techniques, such as ultrasonic dispersion or high-shear mixing, are often employed to improve the distribution of MOFs within the polymer, minimizing aggregation and enhancing the composite’s thermal and mechanical properties [115]. Additionally, physical blending offers flexibility in choosing MOF–polymer combinations, allowing for fine-tuning of composite properties according to specific heat exchanger applications.

Surface functionalization enhances compatibility and bonding between MOFs and polymer matrices by introducing functional groups like -NH_2_ or -COOH. This strengthens the interface, improves mechanical properties, and reduces thermal resistance, optimizing thermal transport in the composite [116]. For metal matrices, surface functionalization can involve coating the MOF particles with metal-compatible agents, which encourages better integration and reduces particle agglomeration within the metal host. This method has been shown to significantly enhance the thermal conductivity and stability of MOF–metal composites under high-temperature conditions. Studies have demonstrated that MOFs like MIL-101 and UiO-66, when functionalized and embedded in aluminum or copper matrices, exhibit superior thermal performance and robustness, making them highly suitable for heat exchanger applications [117].

Electrochemical deposition is a more specialized method used primarily for embedding MOFs in metal matrices. In this process, a metal or alloy is electrodeposited onto a surface along with MOF particles in a controlled environment, ensuring uniform distribution and integration. Electrochemical deposition creates a composite with excellent interfacial adhesion and controlled MOF loading, which is beneficial for enhancing thermal pathways within the material. This method is advantageous for applications requiring precise control over the composite’s microstructure, and it has been successfully applied in embedding MOFs like ZIF-8 within copper and aluminum matrices for enhanced thermal conductivity in heat exchangers [118,119,120].

Each method for embedding MOFs in metal and polymer matrices has unique advantages and challenges. In situ growth ensures strong interfacial bonding but requires precise control of MOF growth conditions, while physical blending offers simplicity but may suffer from non-uniform distribution. Surface functionalization enhances compatibility and integration, especially in polymer matrices, but adds complexity in terms of processing. Electrochemical deposition, though specialized, allows for precise structural control in metal matrices. By carefully selecting and optimizing these embedding techniques, researchers can develop MOF-based composites that meet the rigorous thermal and mechanical demands of advanced heat exchangers [121,122,123].

### 2.2. MOF–Polymer and MOF–Metal Nanocomposites

#### 2.2.1. Advantages and Applications of MOF–Polymer Composites

The integration of MOFs with polymers has shown substantial potential in enhancing the efficiency and performance of heat exchangers, particularly for compact systems like HVAC units. The combination leverages the inherent benefits of polymers, such as flexibility, lightweight structure, and mechanical robustness, alongside the superior surface area and thermal properties of MOFs [124]. This synergy not only improves thermal conductivity but also enhances moisture management, making these composites highly efficient for energy-intensive applications [125]. MOF–polymer composites, such as those involving ZIF-8 embedded within polyimides (PI), have been extensively studied for their enhanced heat transfer capabilities. ZIF-8 is known for its high porosity, stability, and large surface area, which allows for efficient heat dissipation. When ZIF-8 is incorporated into polyimide matrices, it significantly improves thermal conductivity while preserving the mechanical flexibility of the polymer [126]. Some of the prominent MOF–Polymer composites are listed in Table 2, along with their applications.

#### 2.2.2. Applications and Advantages of MOF–Metal Composites

MOF–metal nanocomposites have gained significant attention for their enhanced thermal conductivity and mechanical durability, making them ideal for applications where efficient heat transfer is crucial. By combining MOFs with high-conductivity metals like copper and aluminum, researchers have developed composites that outperform traditional materials in heat exchanger and cooling systems [142,143].

Copper (Cu) is widely recognized for its excellent thermal conductivity (~400 W/m·K), making it an ideal candidate for integration with MOFs to improve heat transfer performance. Studies have shown that embedding MOFs like HKUST-1 (a copper-based MOF) into copper matrices results in composites with enhanced thermal transport properties [144]. The combination of aluminum with MOFs such as MIL-101(Cr) or UiO-66 results in materials that not only enhance heat transfer but also exhibit increased resistance to corrosion and thermal degradation [142]. This improvement is attributed to the high surface area and porosity of the MOF, which facilitates faster heat dissipation, especially in compact systems like HVAC units. Some of the relevant case studies related to heat transfer enhancement using MOFs are noted in Table 3.

### 2.3. MOF-Based Nanomaterials for Enhanced Fouling Resistance

Fouling is a persistent issue in heat exchangers, as the accumulation of unwanted deposits on surfaces reduces heat transfer efficiency, increases energy consumption, and leads to higher maintenance costs. Recent advancements in materials science have shown that coating heat exchanger surfaces with MOFs can mitigate fouling, especially in systems where water or other fluids are involved [153]. The unique structural properties of MOFs, such as their high surface area, tunable pore sizes, and selective adsorption capabilities, make them effective in minimizing fouling, thereby enhancing the efficiency and longevity of heat exchanger systems [154].

Hydrophobic MOFs, such as ZIF-8, have been particularly effective in reducing fouling by repelling water and other contaminants. The hydrophobic nature of ZIF-8 helps in preventing moisture-induced biofilm formation, which is a common problem in condenser units. Some of the studies related to fouling reduced by using MOFs are discussed in Table 4.

### 2.4. Applications in Specific Heat Exchanger Designs

#### 2.4.1. Compact Plate Heat Exchanger

The compact design of plate heat exchangers (PHEs) is highly advantageous for applications demanding space efficiency, such as HVAC systems, automotive cooling, and chemical processing units. These exchangers consist of closely packed plates that maximize the surface area for heat transfer; enhancing the overall efficiency while minimizing space requirements remains a significant challenge, leading to decreased thermal performance and increased maintenance costs [159]. In recent years, utilization of MOFs in the design of heat exchangers has demonstrated promising results in enhancing heat transfer efficiency and reducing fouling [160]. PHEs consist of corrugated stainless steel plates sealed with gaskets to prevent fluid mixing and ensure efficient heat transfer via a counter-current flow design. Their modular construction allows thermal output adjustment, easy maintenance, and cleaning, making them ideal for industries like food processing, pharmaceuticals, shipbuilding, and oil and gas [159,161]. ZIF-8 MOFs have demonstrated a substantial improvement in thermal performance due to their ability to facilitate efficient thermal transport and fluid flow.

A study reported by Zhang et al. demonstrated ZIF-8/PI coated heat exchanger plates could achieve a significant enhancement in heat transfer rates while also reducing fouling due to the hydrophobic characteristics of the composite. Additionally, an experimental investigation revealed a compact PHE with a ZIF-8 composite led to a 12% increase in heat transfer efficiency compared to uncoated units. This improvement is attributed to the unique structure of ZIF-8 reducing surface energy, thus minimizing the adhesion of fouling agents [162]. Table 5 discusses some research studies, along with their key findings, related to PHE employing MOF as coating material.

#### 2.4.2. Fin Tube Heat Exchangers

The integration of MOFs in fin tube heat exchanger designs offers considerable promise, especially in high-temperature and high-pressure environments. Fin tube exchangers are commonly used in industrial settings due to their ability to increase heat transfer surfaces, thereby enhancing efficiency. By embedding MOF–metal nanocomposites, such as those made with copper or aluminum, these heat exchangers can achieve even greater thermal performance while maintaining structural integrity under challenging conditions [160].

Sun et al. [163] carried out an experimental study under outdoor conditions in Singapore. The system achieved a moisture removal rate of 9.32 g/kg of dry air, with a thermal COP of 0.70 and a waste heat utilization efficiency of approximately 74.2%. To further boost energy efficiency, a heat recovery subsystem was implemented to capture waste heat from the exhausted regeneration air. By optimizing the pre-regeneration process to 3 min, the system’s moisture removal was slightly reduced to 9.01 g/kg; however, this adjustment significantly enhanced the thermal COP and waste heat utilization to 1.34% and 86.5%, respectively. Similarly, an another study demonstrated that coating aluminum fins with CPO-27 (Ni) resulted in a 15% increase in heat transfer efficiency due to improved thermal conductivity and enhanced fluid flow across the heat exchanger surfaces. The porous nature of MOFs contributes to effective heat dissipation by reducing thermal resistance while maintaining excellent mechanical stability, even at high operating temperatures [164]. Table 6 discusses some research studies, along with their key findings, related to fin tube heat exchangers employing MOF as a coating material.

#### 2.4.3. Wire-Finned Tube Heat Exchangers

The use of Metal–Organic Frameworks (MOFs) in wire-finned tube heat exchangers for HVAC and refrigeration systems has gained attention due to their exceptional moisture adsorption and desorption properties. MOFs are highly porous materials with large surface areas and tunable pore sizes, which make them highly effective in enhancing the efficiency of dehumidification processes, thus improving overall system performance [160]. Some of the studies found in the literature are discussed in Table 7, along with their key findings.

## 3. Innovations and Challenges in MOF-Based Heat Exchanger Applications

### 3.1. Innovations in Synthesis and Fabrication

In recent years, significant innovations in the synthesis of MOF-based nanocomposites have focused on improving scalability and cost-effectiveness, crucial for their industrial adoption.

#### 3.1.1. Scalable Synthesis Method

Traditional synthesis methods, such as solvothermal processes, have been widely used to produce MOFs, but their scalability has often been limited by high energy consumption and lengthy reaction times [171]. To address these limitations, microwave-assisted synthesis has emerged as a promising alternative. This method accelerates reaction times and offers better control over particle size and morphology, resulting in high-quality MOFs with improved performance in heat exchanger applications [172,173].

Solvothermal methods, though widely employed for laboratory-scale production, have seen advancements in recent years to improve scalability. Researchers have developed optimized protocols to enhance the synthesis efficiency and yield, making solvothermal processes more suitable for large-scale production [174]. This advancement, coupled with the development of new MOF precursors and solvents, has allowed for a more controlled and reproducible production process, enabling MOFs to be produced at a commercial scale without compromising material quality [175,176].

**Figure 8:**
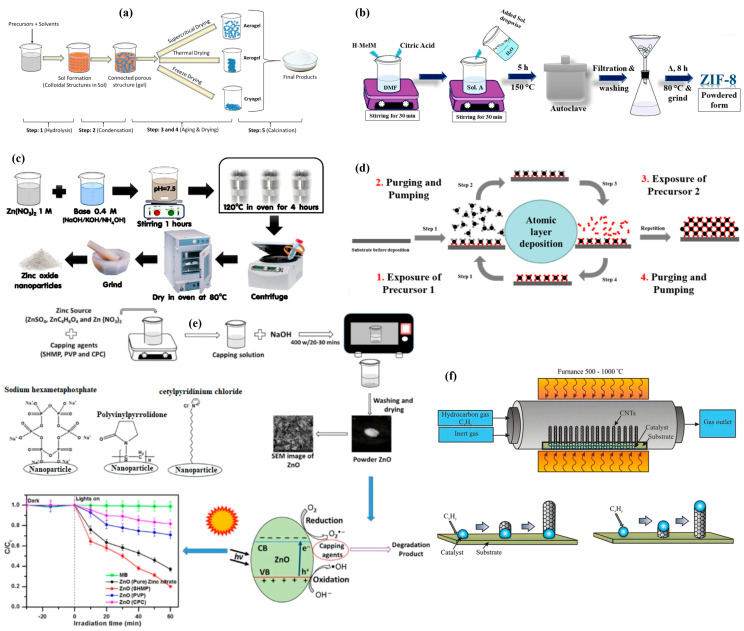
Operating principles for (**a**) sol–gel process to synthesize metal oxide nanoparticles [181], (**b**) solvothermal synthesis process for MOF (ZIF-8) [182], (**c**) hydrothermal process for synthesis of metal oxide (ZnO) nanoparticles [183], (**d**) atomic layer deposition (ALD) synthesis [184], (**e**) microwave-assisted synthesis of metal oxide nanoparticles [185], and (**f**) chemical vapor deposition (CVD) synthesis of CNTs along with their base and tip growth mechanism [186] (reprinted with permission from Springer Nature, Wiley, and MDPI).

Microwave-assisted synthesis has revolutionized the production of metal–organic frameworks (MOFs), making it more cost-effective and efficient, especially for industrial applications [177]. Traditionally, the synthesis of MOFs using solvothermal methods is time-consuming, often requiring several hours to days, along with significant energy consumption [178]. In contrast, microwave-assisted synthesis dramatically reduces reaction times to just a few minutes, while also lowering the energy input, making it a more sustainable and economically viable method for large-scale production provided by microwave radiation, which leads to uniform nucleation and accelerated crystallization rates, resulting in MOFs with consistent particle size and morphology, and enhanced crystalline characteristics. This uniformity is crucial for industrial applications where consistent material properties are needed to ensure reliable performance, especially in heat exchanger systems where thermal conductivity and adsorption capacity are critical [179,180]. Figure 8 represents the schematics for the operating principles of the widely used synthesis methods for nanoparticles.

#### 3.1.2. Surface Functionalization Method

**Figure 9:**
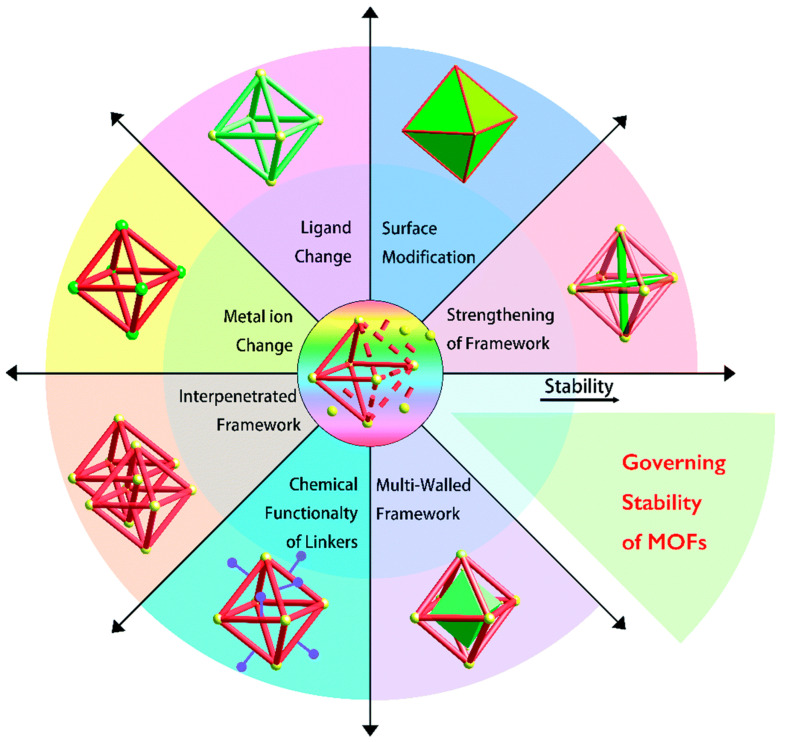
Various techniques used to attain stability of MOFs [190] (reprinted with permission from Royal Society of Chemistry).

Surface functionalization of MOFs has gained significant attention as a means to enhance their properties for heat exchanger applications. By grafting specific functional groups onto the surface of MOFs, researchers can improve thermal conductivity, mechanical strength, and chemical stability, making these materials more suitable for demanding industrial environments [187]. Such techniques can be broadly categorized into structural modifications and post-synthetic functionalization techniques. Techniques such as ligand exchange, metal ion exchange, and annealing can improve stability by strengthening the coordination bonds or modifying the MOF’s surface properties [188]. For instance, ligand exchange with hydrophobic molecules has been shown to protect MOFs against moisture degradation [189]. Figure 9 represents several strategies through which properties of MOFs can be tailored to attain stability.

Functionalization with alkyl or amine groups has been shown to enhance thermal conductivity by creating better interfaces between the MOF particles and the surrounding matrix. In composite material, ZIF-8 functionalized with amine groups exhibited better integration into polymer matrices, leading to increased mechanical strength and durability, which are essential for heat exchanger components that operate under variable thermal cycles [191]. Table 8 discusses some of the research studies carried out for enhancement in thermal properties of MOFs using the Surface Functionalization Method.

### 3.2. Challenges in Industrial Integration

#### 3.2.1. Cost and Feasibility

Integrating MOFs into industrial applications, particularly in heat exchange systems, faces significant challenges, with cost being one of the most critical barriers. The synthesis, fabrication, and incorporation of MOFs into large-scale industrial processes remain expensive, largely due to the complex and energy-intensive methods traditionally used, such as solvothermal and hydrothermal synthesis. These methods require high temperatures and prolonged reaction times, leading to increased production costs [196]. Additionally, the precursor materials for MOFs, especially metals and organic linkers, are not always readily available with the desired stability, porosity, and mechanical robustness to withstand industrial conditions, such as high pressure and temperature, further driving up costs. For instance, integrating MOFs into polymer or metal matrices to enhance their thermal and mechanical properties involves additional steps like surface functionalization, which can increase both material and labor expenses [197]

Severino et al. [198] investigated the feasibility of using safe and economical batch production techniques to scale up the synthesis of MOF MIL-160(Al) for industrial applications. They developed a straightforward approach to estimate the production cost of this MOF, utilizing data gathered from laboratory pilot-scale experiments. This cost evaluation, for the first time, considered all critical process factors, including production scale, raw material expenses, recirculation, and washing procedures. According to their findings, the projected production costs could range from approximately USD 55 per kg for an annual output of 100 tons to about USD 29.5 per kg if scaled up to 1000 tons per year. Similarly, Shahvari et al. [199] explored the impact of substituting traditional solid sorbent materials like silica gel with various metal–organic frameworks (MOFs). Their study revealed that the top-performing MOFs among those tested—specifically, Co_2_Cl_2_ (BTDD) (Co), Aluminum fumarate, and CAU-23 (Al)—could be regenerated at significantly lower temperatures, ranging between 40 and 60 °C, depending on ambient conditions. This is a notable improvement over silica gel, which typically requires regeneration temperatures between 80 and 140 °C. Furthermore, the energy consumption for a MOF-based sorbent wheel was found to be substantially lower, ranging from half to as little as one-eighth of that needed for systems utilizing silica gel, varying with environmental conditions.

Cost–benefit analyses in the literature indicate that the initial capital expenditures are high, yet the long-term operational savings can justify the investment. For example, research discussed earlier reduced energy consumption up to 50%, resulting in substantial cost savings over time. As the technology scales and newer, more efficient synthesis methods, such as microwave-assisted synthesis, become more prevalent, the costs associated with MOF production are expected to decrease, potentially making industrial integration more feasible [200].

#### 3.2.2. Long-Term Stability and Durability

Ensuring the long-term stability and durability of MOF-based nanocomposites under real-world operational conditions remains a significant challenge, particularly in harsh environments such as high temperatures, elevated pressures, and corrosive atmospheres. These conditions can degrade the structural integrity of MOFs, potentially reducing their performance over time. The inherent porous structures of MOFs, while advantageous for adsorption and heat transfer applications, are also prone to collapse or degradation when exposed to extreme conditions [201]. Addressing these challenges is essential for their practical use in industrial systems like heat exchangers and HVAC systems.

Recent studies have demonstrated efforts to enhance the stability of MOFs through surface modifications and structural tuning. Cai et al. found that the incorporation of stabilizing agents, such as graphene oxide, into MOF composites like MOF-808/GO resulted in enhanced resistance to thermal and mechanical stress. This hybrid structure improved the overall durability of the composite, particularly under conditions of fluctuating humidity and high heat [202]. Similarly, HKUST-1, while widely used due to its high surface area, suffers from instability in humid conditions, as water molecules can disrupt its metal–ligand bonds [203]. Research has proved that introducing hydrophobic functional groups or creating composite structures with polymers provides additional stability [204].

Despite these advancements, research is needed to develop MOFs that can withstand prolonged exposure to harsh conditions without compromising their adsorption capacity or thermal properties. There is ongoing work in the area of composite materials, where embedding MOFs into metal or polymer matrices helps in enhancing their structural integrity while retaining their desirable properties. These hybrid composites show promise in extending the operational life of MOFs in demanding applications, but scalability and cost remain barriers that must be addressed for widespread industrial adoption.

## 4. Research Gaps and Future Directions for MOF-Based Nanocomposites in Heat Exchangers

Advancements in material selection can lead to substantial improvements in performance across various applications. For Desiccant-Coated Heat Exchanger (DCHE) systems, the ideal desiccant should possess a high water adsorption isotherm, rapid water uptake kinetics, and regeneration temperatures close to or below room temperature. These properties help minimize the need for excess heat during desiccant regeneration, even after numerous operational cycles, ensuring energy efficiency in DCHE applications [205,206,207]. Moreover, the desiccant should be cost-effective, environmentally safe, and support recycling for sustainable use. Early materials like silica gel, activated alumina, and AC were widely used due to their high surface areas and effective water adsorption characteristics. To further enhance performance, composites were developed by using these porous materials as a foundational matrix, infused with hygroscopic salts, significantly boosting their adsorption capacity—often doubling or even quadrupling it [208,209]. However, increasing the salt content may lead to challenges such as corrosion and deliquescence, limiting the effectiveness of hygroscopic salt impregnation. This drawback did not hinder further research, which eventually led to the development of more advanced materials like MOFs. MOFs can be precisely engineered for specific properties, offering significant advantages. Yet, their high costs currently prevent widespread commercial use for large-scale applications. Nonetheless, advancements in the commercialization process and scaling up production could reduce costs, enabling efficient, high-speed manufacturing that would make MOFs more economically viable [196,210].

### 4.1. Multifunctional MOFs

The development of multifunctional MOF-based composites by integrating them with advanced materials, such as CNT and GO, represents a promising frontier in heat exchanger technology. These hybrid nanocomposites offer a combination of enhanced properties like superior heat transfer, efficient gas adsorption, and catalytic activity, making them ideal for next-generation heat exchangers. Due to their inherent characteristics of both MOFs and carbon-based materials, researchers aim to achieve optimal thermal conductivity, structural stability, and adsorption capacity [211].

For instance, incorporating CNTs into MOFs can significantly improve their thermal conductivity. A study demonstrated that combining CNTs with MOFs such as HKUST-1 resulted in enhanced heat transfer efficiency due to the high thermal conductivity of CNTs, which compensates for the low thermal conductivity typically associated with pure MOFs. Furthermore, MOF composites have shown superior thermal stability and mechanical strength, crucial for use in harsh industrial environments. Research involving MOF-5 combined with reduced graphene oxide (rGO) reported improved adsorption performance and heat transfer efficiency, making it suitable for energy-efficient HVAC systems [212]

These hybrid composites are multifunctional beyond heat transfer. For example, integrating MOF-74 with graphene oxide has been explored for applications that require both gas adsorption and thermal management [213]. This combination allows for effective heat dissipation while maintaining high adsorption capacity, which is advantageous in heat exchangers used for gas separation processes. Additionally, functionalizing MOFs with conductive materials enhances their electrical conductivity, which can be beneficial in thermoelectric applications [214]. Table 9 represents some multifunctional MOFs, along with their enhanced properties and applications.

Despite the promising performance of these multifunctional composites, challenges such as scalability, cost, and long-term stability under operational conditions remain. However, with continued research and the optimization of synthesis methods, the integration of MOFs with advanced materials could lead to the next generation of efficient and sustainable heat exchanger systems. As these hybrid materials continue to be tested and refined, they hold the promise of revolutionizing not only heat exchangers but also broader energy management and environmental technologies.

### 4.2. Bridging Gaps Between Research and Industry

Collaboration between academia and industry is crucial in advancing the field of MOFs research and ensuring that breakthroughs in laboratory settings translate into real-world applications. While academia excels in exploring the fundamental science behind MOFs, industry partners can bring the practical expertise needed for large-scale manufacturing, commercialization, and integration into existing systems. The synergy between these two sectors can accelerate the development of MOF technologies, making them more accessible and applicable for industrial processes like heat exchangers, gas storage, and separation.

One of BASF’s recent innovations focuses on adsorptive dehumidification in air conditioning systems. Traditional air conditioners are tasked not only with cooling incoming air but also with controlling its humidity levels. Typically, these systems cool the air to a set temperature, causing moisture to condense. This dew pointing of air is highly energy-intensive and can account for up to 60% of the total energy usage of the unit, depending on environmental conditions. A more energy-efficient approach involves utilizing an adsorption-based system where moisture is selectively captured by a MOF. This method significantly lowers the electricity demand of air conditioning units. Under optimal conditions, using MOFs for air drying could reduce energy consumption by 50–60%, which translates to thousands of kWh savings annually for a standard office building. Compared to traditional materials like silica gel, MOFs offer 1.5 to 2 times higher volumetric energy density, allowing for more compact device designs, and provide a 27% improvement in the coefficient of performance. This not only enhances efficiency but also supports downsizing the equipment while achieving superior energy conservation [229]

However, adsorption systems come with their own energy costs. Once the MOF becomes saturated with moisture, it requires a desorption phase to regenerate the material and release the adsorbed water. This regeneration process can utilize thermal energy sourced from low-temperature heat supplies, such as solar thermal systems or waste heat produced by the air conditioner’s own compressor. Such applications demonstrate that the distinctive characteristics of MOFs outperform those of current materials and technologies. As new use cases emerge, and production scales up, costs are expected to decline, making these materials more viable for broader applications. The continued reduction in expenses due to economies of scale will facilitate their integration into additional areas, further expanding their practical uses.

NuMat Technologies is a pioneer in the commercialization of MOF-based solutions, focusing on gas storage and separation technologies [230]. Their innovative systems utilize MOFs with high surface areas and tunable pore structures to store gases like methane and hydrogen efficiently. This makes them ideal for use in fuel cell vehicles and portable energy devices, where compact and lightweight storage is critical. NuMat Technologies has not only advanced MOF-based gas storage systems but has also addressed the challenges of cost-effectiveness and scalable production. By leveraging computational design and automation, NuMat reduces the time and expense associated with MOF synthesis, enabling large-scale production without compromising quality. Their manufacturing processes are optimized for industrial scalability, making MOFs more accessible for commercial applications. The cost-effectiveness of NuMat’s technology is further highlighted in applications like hydrogen and methane storage for fuel cell vehicles, where lightweight and compact systems are essential. MOFs provide higher storage capacities compared to traditional materials, reducing the overall system size and cost. Additionally, NuMat’s focus on modular production techniques ensures flexibility and adaptability for different industries [229]. These advancements underline how MOFs can transition from laboratory-scale research to practical, economically viable solutions, paving the way for widespread adoption in clean energy, gas separation, and other industrial applications.

MOFs could soon compete with established materials like activated carbons, zeolites, and silica in terms of cost and performance. At the very least, they may serve as crucial components in multi-material systems, such as polishing layers, depending on the specific application. Achieving this will require specialized expertise and robust supply chains to seize emerging opportunities effectively. In the long run, MOFs have the potential to become as widespread as plastics, becoming an essential part of various industries, products, and everyday life in the 21st century. Although it may take decades to see if this forecast fully materializes, it is clear that MOFs have already moved beyond purely academic research. The focus now is no longer on whether these materials will find commercial success, but rather on “what’s next” and which players are best poised to leverage the emerging opportunities in this field.

### 4.3. Green and Pollution-Free Preparation Methods for MOFs

The development of environmentally friendly and sustainable synthesis methods for MOFs has gained increasing attention in recent years. Traditional MOF synthesis methods, such as solvothermal and hydrothermal processes, often involve the use of toxic solvents, high energy consumption, and significant waste generation [231]. To address these issues, researchers are exploring alternative green synthesis approaches that minimize environmental impact while maintaining material performance [232].

- One promising approach is solvent-free synthesis, which eliminates the use of hazardous organic solvents by employing mechanochemical methods [233]. In this process, the metal precursors and organic linkers are ground together in a ball mill, enabling the formation of MOFs under ambient conditions. This method not only reduces waste but also significantly lowers energy consumption, making it a highly sustainable alternative [234].
- Another green technique is the use of water as a reaction medium. Aqueous-based synthesis methods utilize water as a benign solvent, reducing the ecological footprint associated with solvent disposal [235]. For instance, the production of MIL-53 [236] and MIL-100/101 [237] has been successfully demonstrated using water as the primary solvent, achieving comparable performance to their traditionally synthesized counterparts.
- Microwave-assisted synthesis is another eco-friendly method that offers rapid reaction times and reduced energy requirements [172]. This technique utilizes microwave irradiation to heat the reaction mixture uniformly, leading to faster crystallization and higher yields [238]. Moreover, the process can often be conducted in water or ethanol, further reducing the environmental impact.
- Lastly, the adoption of waste valorization strategies, such as using industrial by-products as precursors for MOF synthesis, exemplifies a circular economy approach [239]. These strategies not only reduce the cost of raw materials but also contribute to waste reduction and resource conservation [240].

Incorporating these green synthesis methods into MOF production processes holds great promise for enhancing the sustainability of MOFs in heat exchanger applications, aligning with global efforts to reduce environmental pollution and promote sustainable industrial practices.

## 5. Conclusions

This review has provided an in-depth analysis of the current landscape, innovations, and future directions for the application of MOFs in heat exchangers. As highlighted throughout, MOFs hold immense promise for enhancing the efficiency and sustainability of heat exchange systems due to their tunable porosity, high surface area, and exceptional adsorption and thermal properties. However, realizing their full potential in real-world applications requires addressing several key challenges, from cost-effective synthesis to maintaining stability under harsh operational conditions. In this work, the authors have provided a comprehensive review on the applications, innovations, challenges, and future directions of MOFs in heat exchangers.

- MOFs integrated into a heat exchanger, such as wire-finned and shell-and-tube designs, have shown remarkable gains in heat transfer rates and moisture adsorption, and reduction in fouling resistance. Studies have proven that MOF-based coatings, like MOF-303/GO and HKUST-1 composites, enhance both water uptake, up to 22%, and thermal diffusivity, outperforming traditional materials such as silica gels and activated alumina. MOFs also outperformed traditional materials such as silica gel by enhancing COP as much as six times in comparison to it.
- This research also focused on the innovations and challenges surrounding MOF-based heat exchanger applications. Innovations in scalable synthesis methods, such as microwave-assisted synthesis and surface functionalization techniques, are key to reducing costs while ensuring consistent material quality. These advancements have the potential to make MOFs more economically viable for large-scale industrial applications. Furthermore, surface functionalization has been shown to enhance the thermal conductivity, mechanical stability, and chemical resilience of MOFs, making them more suitable for demanding environments. Yet, issues such as long-term stability under high temperatures, pressure, and corrosive conditions still need to be addressed, as shown by ongoing research into the durability of various MOF composites under operational stresses.
- The research gaps and future directions for MOF-based nanocomposites in heat exchanger systems are also studied in this research. The integration of multifunctional MOF hybrids, combining materials like GO, AC, and CNT, presents a promising path forward for developing heat exchangers with enhanced performance across multiple domains, such as heat transfer, gas adsorption, and catalysis. The multifunctional MOFs of CNT enhanced the thermal conductivity up to seven times in comparison to the actual MOFs. However, these hybrid solutions will require further collaboration between academia and industry to optimize material properties and develop scalable production processes.
- Real-world industrial case studies have also been analyzed such as BASF’s innovation in adsorptive dehumidification, which leverages MOFs to reduce energy consumption in air conditioning systems by 50–60%, offering higher volumetric energy density and a 27% improvement in COP compared to silica gel. While MOFs require energy for regeneration, this can be sourced from low-temperature heat like solar or waste heat. Scaling production is expected to lower costs, broadening their applicability and enhancing energy efficiency. Similarly, NuMat Technologies exemplifies the transition of MOFs from research to industrial applications by developing cost-effective, scalable production methods for gas storage systems. Their innovations demonstrate the potential of MOFs in clean energy and industrial processes, emphasizing their role in advancing sustainable and economically viable solutions.
- Green synthesis methods for MOFs include solvent-free mechanochemical techniques, which eliminate hazardous solvents and reduce energy consumption by grinding metal precursors and linkers. Aqueous-based synthesis uses water as a benign solvent, lowering environmental impact. Methods for production of MIL-53 and MIL-100/101 using water as the primary solvent have displayed commendable performance in comparison to traditional synthesis counterparts. Microwave-assisted synthesis offers rapid, energy-efficient crystallization, often using eco-friendly solvents.

Overcoming challenges related to synthesis costs, material durability, and integration into existing industrial systems is essential to fully harness the potential of MOF-based technologies. Advances in scalable synthesis techniques, computational modeling, and collaborative efforts between academia and industry pave the way for MOFs to revolutionize heat exchanger efficiency, promoting energy conservation and sustainability. Ongoing research focused on improving stability and lowering production costs will be pivotal in establishing MOFs as a widely adopted solution for energy-efficient thermal management systems. Moreover, embedding MOF-based systems with IoT sensors and advanced monitoring tools can further optimize real-time performance and ensure efficient operation.

## Figures and Tables

**Table 1:** Thermal conductivity values of various types of MOFs, as determined in different studies.

S. No.	MOF	Thermal Conductivity in W/(mK)	Main Findings	Ref.
1	Aluminum Fumarate	0.07	Silres^®^ was used as a binder in thicknesses from 140 to 610 μm, and it was extensively studied at 30–60 °C. Aluminum sample plates coated with aluminum fumarate and a silicon binder demonstrated conduction heat transfer mechanisms within the coating layer (0.07 W/mK). The samples exhibited consistently high performance.	[98]
2	Cu-BTC UiO-66 UiO-67	0.39 0.11 0.19	Increasing the pore size leads to higher thermal conductivity, as it reduces the number of resistive junctions per unit length. The inter-particle contact conductance values for UiO-66, UiO-67, and Cu-BTC are 0.0117 μW/K, 0.09 μW/K, and 0.82 μW/K, respectively.	[99]
3	HKUST-1	0.44–0.73	The thermal conductivity of HKUST-1 is reduced by 40–80%, influenced by the presence of adsorbates such as water, methanol, or ethanol.	[100]
4	Idealized MOF structures featuring various pore shapes, including cubic pores, triangular channels, and hexagonal channels	0.03	MOFs with smaller pores exhibit lower thermal conductivity due to phonon scattering caused by interactions between gas molecules and the crystal structure. Conversely, in larger pores (over 1.7 nm), the presence of adsorbed gas has minimal impact on thermal conductivity.	[101]
5	MIL-101/20% Few Layer Graphene (FLG)	0.8322–0.8603	These observations were made at 30–130 °C. Observed thermal conductivities were 5.79–6.54 times that of pure MIL-101.	[102]
6	MOF-5	0.1218–0.1477	In this study, the transient plane source technique was used. The specific heat capacities of pure MOF-5 at low temperatures were observed for a range between 93 and 313 K.	[103]
7	MOF-5	0.32	The value was determined at 300 K. The thermal conductivity increases with temperature in the range of 6–300 K, reaching a peak around 20 K.	[104]
8	MOF-5	0.33	They observed that the extremely low thermal conductivity is attributed to intense phonon–phonon scattering caused by the dense and interwoven low-frequency phonons.	[105]
9	Ni_3_(2,3,6,7,10,11-hexaiminotriphenylene)_2_ (Ni_3_(HITP)_2_) a 2D MOF	0.21	The steady-state method was used in this research study. The ultralow thermal conductivity is attributed to several factors: (a) phonons, which represent lattice vibrations; (b) the heterogeneity of atomic masses and bond stiffness, which leads to phonon scattering; (c) the disordered arrangement of individual layers, resulting in additional phonon scattering; and (d) the very small particle size of crystallites and grain boundaries, which further contribute to phonon scattering.	[106]
10	Tetracyanoquinodimethane (TCNQ) @HKUST-1	0.23–0.31	The incorporation of TCNQ enhances thermal conductivity by increasing the phonon density in the low-frequency/high-group-velocity range.	[107]
11	ZIF-8	0.165–0190	With the temperature increment from 300 to 1000 K. In ZIF-8, longitudinal vibrations account for 60% of thermal transport, while transverse vibrations contribute 40%.	[108]
12	ZIF-8(H)ZIF-8 (Cl)ZIF-8 (Br)ZIF-8 (CH3)	0.165 0.138 0.142 0.174	The presence of functional groups such as -H, -CH3, -Cl, and -Br leads to a decrease in thermal conductivity, primarily due to the damping effect caused by acoustic mismatch.	[109]
13	ZIF-8	0324–0.328	In this study, the transient plane source technique was used. Thermal conductivity remains nearly constant regardless of the ambient gas and pressure, ranging from atmospheric pressure to a vacuum.	[110]

**Table 2:** Prominent MOF–Polymer Composites and their applications in different fields.

S. No.	Composite Name	Description	Applications	Ref.
1	ZIF-8/Polyimide (PI) Composite	ZIF-8 embedded in polyimide enhances thermal conductivity while maintaining the flexibility and robustness of the polymer.	HVAC systems for efficient heat transfer and moisture management, as well as in gas separation applications.	[127,128,129]
2	MIL-101(Cr)/Polysulfone (PSU) Composite	MIL-101(Cr) combined with PSU improves mechanical strength and thermal stability. The high surface area of MIL-101(Cr) contributes to superior adsorption capabilities.	Air purification, nano-filtration, and gas separation.	[130,131,132]
3	UiO-66/Polyethylene Oxide (PEO) Composite	UiO-66 incorporated into PEO enhances thermal conductivity and mechanical properties, along with increasing moisture resistance.	Lithium-ion batteries, proton exchange membranes, drug delivery, and water filtration systems.	[133,134,135]
4	MOF-5/Polymethyl Methacrylate (PMMA) Composite	MOF-5 embedded in PMMA improves the thermal and mechanical properties of the polymer, enhancing its use in structural applications.	Lightweight thermal insulation panels and coatings.	[136,137]
5	HKUST-1/PDMS Composite	HKUST-1 integrated with PDMS increases thermal conductivity while retaining the flexibility and elasticity of PDMS.	Heat exchangers, wearable electronics, and gas sensors.	[138,139]
6	MOF-801/Polyurethane (PU) Composite	MOF-801 embedded in PU improves thermal stability and adsorption properties, making it ideal for applications requiring flexibility and durability.	HVAC systems, energy-efficient coatings, and adsorptive cooling systems.	[140,141]

**Table 3:** Relevant research studies in which MOFs are used for enhancement of heat transfer.

S. No.	MOF Used	Heat Exchanger Type	Main Findings	Ref.
1	Sodium polyacrylate	Single-row finned-tube	Exchanger total fin area was 7.26 m^2^. Regeneration temperature of 50 °C was used. Cycle Time in this study was 15 min. Obtained Coefficient of Performance (COP) of 0.294, which was increased by 16.2%.	[145]
2	MIL-100 (Fe)	Rectangular finned heat sinks	Exchanger total fin area was 1.452 m^2^. Regeneration temperature of 50 °C was used. Cycle Time in this study was 15 min. Obtained COP of 0.42, which is an almost 43% enhancement in comparison to [117].	[146]
3	Aluminum Fumarate MIL-100 (Fe) MIL-100 (Fe)/G MOF-303/G MOF-801 MOF-801/G	Wire-finned heat exchanger	Exchanger total fin area was 0.9948 m^2^. Regeneration temperatures of 45, 60, and 70 °C were used. Cycle Time in this study was 15 min. Obtained COP of 0.62, 1.02, and 1.20 at regeneration temperatures of 45, 60, and 70 °C, respectively.	[147]
4	Aluminum Fumarate	Wire-finned heat exchanger	Coated wire-finned heat exchanger produced Specific Cooling Power (SCP) of 682 W/kg. Obtained COP of 0.32, which is almost 39% more than packed exchanger.	[13]
5	Aluminum Fumarate	Packed heat exchanger	The optimal half-cycle time was observed at 250 s, with the temperatures of the evaporator, condenser, adsorption, and desorption water at 24 °C, 25 °C, 25 °C, and 85 °C, respectively. Under these conditions, the SCP, Specific Desorption Water Production (SDWP), and COP were 226 W/kg, 8.66 L/kg/day, and 0.5, respectively.	[148]
6	MIL-100 (Fe)	Packed heat exchanger	The MOF-coated heat exchangers delivered a cooling power density of 82 W·L^−1^. The results show that the system achieved a high COP of up to 7.9 and reduced energy consumption by 36.1% compared to the conventional Vapor-Compression Air Conditioning (VCAC) system with reheating.	[51]
7	CAU-23 CAU-10 Co_2_C_l2_ (BTDD)	Plate-type heat exchanger	It was observed that MOF-based systems can achieve a COP that is 2.7 to 6 times higher than silica gel-based systems, with an optimal regeneration temperature range of 40 to 75 °C.	[149]
8	MIL-101 (Cr)	Cross-flow heat exchanger	This study was carried out in the hot and humid climate of Qatar. Throughout the entire cooling season, the hybrid system utilizing MIL-101 (Cr) was found to reduce thermal and electrical energy consumption by 17% and 48%, respectively, compared to the system using silica gel. This resulted in a 27% decrease in operating costs, with a payback period of 11 years, based on the current market price of MIL-101 (Cr).	[150]
9	CPO27 (NI) MIL100 (Fe) MIL-101 (Cr) Aluminum Fumarate silica gel	Cross-flow heat exchanger	The results indicated that Aluminum Fumarate achieved the highest COP of 0.65, with a water removal rate of 12.65 g/kg dry air. In contrast, MIL-101 (Cr) demonstrated the highest moisture removal rate of 15.99 g/kg dry air, but its COP was 0.44 when compared to silica gel and other MOF materials used in the study.	[151]
10	MOF–801 Aluminum Fumarate MIL-100 (Fe)	-	The MOF–801/water pair can achieve a gross temperature lift of approximately 30 K with a COP of 0.4867, outperforming aluminum fumarate with a COP of 0.3903 and MIL-100 (Fe) with a COP of 0.0926.	[152]

**Table 4:** Relevant research studies in which reduction in fouling is observed using MOFs.

S. No.	MOF Used	Main Findings	Ref.
1	ZIF-8@PVDF45 HKUST-1@PVDF45	The coated membranes were experimentally evaluated in an air-to-air membrane enthalpy exchanger. Results showed that the effectiveness increased from 44.4% for the original polyvinylidene fluoride (PVDF45) to 45.9% and 47.7% for the ZIF-8@PVDF45 and HKUST-1@PVDF45 membranes, respectively, at a flow rate of 0.24 L/s. This improvement occurs due to improved hydrophilicity and a higher specific surface area, facilitating better moisture adsorption and transfer. These enhancements optimize the interaction between the air and the membrane, boosting the overall dehumidification and heat exchange efficiency. This study highlights a simple and effective approach to improve the efficiency of polymer membranes in air-to-air enthalpy exchangers, potentially reducing energy use in buildings and lowering greenhouse gas emissions.	[129]
2	MIL-110	MOF-based slippery surface infused with ionic liquid demonstrates exceptional antifouling capabilities. The MOF-based slippery surface exhibited excellent antifouling performance, maintaining effectiveness in a 10-day trial and even under more rigorous conditions in a subsequent 21-day test. The amount of lipopolysaccharide (LPS) adsorbed on the Slippery Lubricant-Infused Porous Surface (SLIPS) was reduced by 50% compared to a plain aluminum sheet.	[155]
3	CuZn-MOF-74 (PBMA)	After submerging the coatings in the local seawater for two months, the untreated side was heavily covered with calcareous tubeworms and barnacles, whereas the prepared coatings showed significantly fewer macrofouling organisms. A UV-curable poly butyl-methacrylate (PBMA) coating incorporating a low concentration of MOF-74 (e.g., 3 wt% to 6 wt%) has proven effective in preventing biofouling.	[156]
4	GO/ZIF-8 MOF	Antifouling tests included Adsorption of Bovine Serum Albumin (BSA) and Anti-Biofouling Activity against Bacteria. All the tests supported that GO/ZIF-8 MOF coating has enhanced antifouling characteristics.	[157]
5	PDMS@MOF@Cu	Due to its super hydrophobic properties, the PDMS@MOF@Cu mesh demonstrates outstanding antifouling and self-cleaning capabilities, effectively resisting contamination from everyday drinks and dirt.	[158]

**Table 5:** Research studies and their key findings related to PHE employing MOF as coating material.

S. No.	MOFs Used	Main Findings	Ref.
1	Aluminum Fumarate	The research explored the absorption of refrigerants on solid absorbents, presenting a potentially eco-friendly solution for HVAC systems. The study utilized frequency response analysis to assess the adsorption dynamics and equilibrium properties of water on MOF-based nanocoatings. Aluminum plates coated with aluminum fumarate and a silicon binder demonstrated conduction-driven heat transfer mechanisms within the coating layer, achieving thermal conductivity close to 0.07 W/(m·K). The calculated effective thermal resistance ranged from 1 to 4 (m^2^·K)/kW. The decrease in thermal resistance is due to the highly porous structure and large surface area of MOFs, which enhance heat transfer by reducing thermal boundary resistance, while the silicon binder ensures uniform particle distribution and better thermal connectivity.	[98]
2	CAU-23 CAU-10 Co_2_C_l2_ (BTDD)	MOF-based systems demonstrate a COP that is 2.7 to 6 times greater than traditional silica gel-based systems, depending on external environmental conditions. These systems perform most efficiently at regeneration temperatures between 40 °C and 75 °C, allowing the utilization of low-temperature heat sources to enhance energy efficiency. The improvement in COP with MOF compared to silica gel is due to MOFs’ higher water adsorption capacity, faster adsorption kinetics, and the ability to regenerate efficiently at lower temperatures, reducing energy consumption.	[149]
3	MIL-100 (Fe)	The humidity pump utilizing MIL-100(Fe) as the desiccant layer demonstrates significantly better dehumidification performance compared to silica gel-based systems. Key performance metrics for the MOF-based pump include a dehumidification rate of 34.9 g/h, a moisture removal efficiency of 1.14 g/Wh, and a dehumidification COP of 0.46. These performance indicators are approximately double those achieved by silica gel-coated systems. The results highlight the enhanced potential of MOF materials in applications requiring efficient humidity control. The superior performance of MOF-based systems arises from their higher adsorption capacity, faster kinetics, and ability to regenerate at lower energy inputs. MIL-100(Fe)’s tailored pore structure and hydrophilic properties allow it to adsorb and release water more efficiently, significantly boosting dehumidification rates, moisture removal efficiency, and dehumidification COP compared to silica gel.	[146]

**Table 6:** Research studies and their key findings related to fin tube heat exchanger employing MOF as coating material.

S. No.	MOFs Used	Main Findings	Ref.
1	MIL-160 (Al)	A computational fluid dynamics (CFD) approach was used to evaluate the performance of a MOF-based heat pump (MOF-HP), establishing interactions among humid air, desiccant material, and the heat sink. The model incorporated fluid flow, heat transfer, and species transport subdomains and was validated against experimental data under various operating conditions. A parametric analysis identified the optimal desiccant thickness for the system, recommending a range of 0.3 to 0.5 mm for improved geometry. The optimized system configuration demonstrated an increase in the coefficient of performance (COP) by 1.51 to 1.59 times compared to the initial design. The improved COP is attributed to the optimized desiccant thickness because it has minimal thermal resistance, which enhances heat and mass transfer efficiency, reducing energy losses and maximizing the adsorption–desorption cycle’s effectiveness. Experimental validation maintained discrepancies within 15%, confirming the accuracy and reliability of the developed model.	[165]
2	MIL-100 (Fe)	The coating’s hygroscopic performance was tested with varying binder types, binder mass concentrations, and MOF mass concentrations. Optimal performance was achieved at 15 wt% PVP and 20 wt% MOF, resulting in a 51.71% improvement over silica coating. The composite adsorbent’s cost was reduced by 78.04% compared to pure MOF fabrication. The moisture removal capacity (MRC) of the DCHE was 6.740 g/kg, 149.63% higher than that of silica coating. The improvement in MRC is due to the synergistic effect of optimized binder and MOF concentrations, enhancing the material’s water adsorption capacity and reducing structural limitations associated with silica coatings.	[166]
3	Aluminum Fumarate	A full-scale functional heat exchanger coated with microporous aluminum fumarate achieved a maximum COP of 0.72 at an evaporator temperature of 22 °C. For data center cooling, operating with heat rejection at 35 °C and delivering useful cold at 18 °C, the system demonstrated an equilibrium cooling COP of 0.69. The increase in COP results from the MOF coating’s enhanced adsorption–desorption efficiency, which improves heat transfer and cooling performance compared to conventional heat exchangers.	[167]
4	Aluminum Fumarate	The binder solution used for coating comprised a 10% binder–solvent mixture and 90% aluminum fumarate as MOF. Increasing the coating thickness from 0.05 mm to 0.2 mm resulted in an average enhancement of 113% in the dehumidification performance. Extending the operating time from 150 s to 300 s led to a 131% improvement in the average dehumidification performance. The enhancements are due to the increased MOF coating thickness and extended operating time, which maximize the material’s adsorption capacity. Additionally, reducing space velocity increases the contact time between air and the MOF, further improving moisture adsorption efficiency.	[168]

**Table 7:** Key results of research studies on using MOFs in Wire-Finned Tube Heat Exchangers.

S. No.	MOFs Used	Main Findings	Ref.
1	Aluminum Fumarate MIL-100 (Fe) MIL-100 (Fe)/G MOF-303/G MOF-801 MOF-801/G	Wire-finned tubes coated with MOF-303/G demonstrated superior performance compared to those coated with Aluminum fumarate, MIL-100(Fe), MIL-100(Fe)/G, MOF-801, and MOF-801/G, achieving a water uptake of 22%. The COP of the multi-tube heat exchanger coated with MOF-303/G was experimentally evaluated and compared to other desiccant-coated heat exchangers. The results indicated that the COP ranged from 0.6264 to 1.02 to 1.2 at regeneration temperatures of 45 °C, 60 °C, and 70 °C, respectively. MOF-303/G exhibited superior water uptake and COP due to its highly porous structure and exceptional hydrophilic properties, which enhance water adsorption efficiency. Additionally, its optimized thermal conductivity ensures effective heat transfer during regeneration, enabling higher COP at lower regeneration temperatures compared to other MOFs.	[147]
2	Aluminum Fumarate	The wire-finned heat exchanger with a specialized coating achieved a Specific Daily Water Production (SDWP) of 23.5 m^3^/ton/day, outperforming the packed heat exchanger, which reached only 12.7 m^3^/ton/day. In adsorption cooling applications, the coated wire-finned heat exchanger demonstrated a SCP of 682 W/kg and a COP of 0.32, significantly higher than the packed heat exchanger, which recorded 318.5 W/kg and a COP of 0.23. Aluminum fumarate demonstrated superior water uptake and COP due to its high surface area and favorable adsorption isotherms, which enhance moisture capture and energy efficiency. Its effective integration into the wire-finned heat exchanger ensures improved heat and mass transfer, resulting in higher SCP and SDWP compared to packed heat exchangers.	[13]
3	Aluminum Fumarate	A heat-powered adsorption heat pump utilizing MOF adsorbent material was applied in a combined desalination and cooling system. The cooling unit generated over 6.9 kW of cooling power, achieving a COP of 0.26. This performance was obtained using a coating thickness of 0.75 mm on the wire-finned tube heat exchanger adsorber beds. Aluminum fumarate’s high adsorption capacity and efficient heat transfer properties contributed to its enhanced performance.	[169]
4	MIL-101 (Cr)	The validated model was employed to examine the impact of fin height, fin spacing, and tube diameter on SCP and SDWP using silica gel. The results demonstrated that reducing the fin height significantly enhanced both SCP and SDWP, reaching peak values of 1.3226 kW/kg and 23 L/kg/day at a fin height of 5 mm. Additionally, decreasing the fin spacing from 11.5 mm to 3.6 mm led to an improvement in SCP and SDWP by approximately 25%. The improvements in SCP and SDWP occurred because reducing the fin height and fin spacing enhanced the heat and mass transfer rates. A lower fin height reduced thermal resistance for adsorption, while closer fin spacing improved airflow distribution, enabling more efficient moisture removal and cooling performance.	[170]

**Table 8:** Several research studies regarding Surface Functionalization Method to improve properties of MOFs.

S. No.	MOF Used	Function Group Added	Main Findings	Ref.
1	UiO-66 (Zr) MIL-125 (Ti)	amino (-NH_2_)	The amino-functionalized MOFs, H_2_N-UiO-66 (Zr) and H_2_N-MIL-125 (Ti), demonstrate high adsorption enthalpies (89.5 and 56.0 kJ/mol, respectively) and exhibit an exceptionally favorable water adsorption isotherm due to their increased hydrophilicity. In particular, H_2_N-MIL-125 shows a notably steep increase in its water adsorption isotherm, making it highly advantageous for heat pump applications.	[77]
2	MIL-101-NH_2_	(Cr)	A new fatty acid@MOF composite phase change material (PCM) has been developed, achieving shape stabilization for the first time. This innovation addresses common challenges associated with traditional PCMs, such as leakage and significant volume changes during phase transitions. The SA@Cr-MIL-101-NH_2_ composite demonstrated excellent thermal stability and long-lasting durability, making it a promising candidate for renewable energy storage applications.	[192]
3	MIL-53	(Al)	The modified MIL-53 (Al) MOFs were found to adjust both hydrophilicity and hydrophobicity characteristics, enhancing water adsorption capacity to as much as 0.9 g/g. The ligand-extended MIL-53 (Al) variants demonstrated superior water transfer efficiency between humid (relative humidity of 80–90%) and regeneration conditions (RH of 30%). They also achieved a substantial Thermal Energy Storage Density (TESD) of up to 1.54 MJ/L.	[193]
4	HKUST-1	(Cu)	In this study, HKUST-1 was successfully grown on copper nanofibers using a homologous Cu self-transformation growth method, resulting in a uniform and tightly bonded MOF coating. The composite retained a high surface area of 1473 m^2^/g. Consequently, the HK@Cu-NF composite, with an 80% loading of HKUST-1, demonstrated thermal diffusivity over three times greater than that of pure HKUST-1.	[194]
5	MIL-125 (Ti)	hydroxyl (-OH), amino (-NH^2^), nitro (-NO_2_), bromo (-Br), and pyridine (-C_5_H_5_N)	The experimental findings indicate that (i) OH-MIL-125(Ti) exhibits adsorption and desorption kinetics that are two to three times faster than those of the unmodified MOF, and (ii) NH_2_-MIL-125(Ti) demonstrates the most favorable water adsorption characteristics, particularly regarding maximum water capacity and efficient water transfer. Additionally, the introduction of the NO_2_ functional group increases the hydrophobic chain length, suggesting its suitability for desiccant-based dehumidification systems.	[195]

**Table 9:** Enhancements in properties of multifunctional MOFs, along with their applications.

S. No.	Multifunctional MOFs	Enhancement in				Applications	Ref.
		Thermal Properties	Mechanical Stability	Electrical Conductivity	Adsorption Characteristics		
1	HKUST-1 with CNT	✔	✔	-	-	Heat Exchangers HVAC	[215]
2	MOF-5 with GO	-	✔	✔	✔	Gas Separation Electrocatalysis	[214]
3	MOF-74(Ni) with GO	-	✔	-	✔	Gas Adsorption and Separation	[216]
4	MIL-101 (Cr) with AC	-	✔	-	✔	Gas Separation Carbon Capture	[217]
5	MIL-100 (Fe) with CNT	✔	✔	-	✔	Gas Separation Wastewater Decontamination	[218,219]
6	MOF-808 with CNT	-	✔	-	✔	Gas Adsorption and Separation Pharmaceuticals Agrochemical Wastewater Treatment	[220,221]
7	MIL-53 with Graphene Oxide	✔	✔	-	✔	Dye Adsorption Photocatalyst	[222,223]
8	UiO-66 with CNT	-	✔	-	✔	Photocatalyst Membranes	[224,225]
9	Aluminum Fumarate	✔	✔	-	✔	Dye Adsorption Pharmaceuticals Membranes	[226,227]
10	UiO-67 with Graphene Oxide	-	✔	-	✔	Electrochemical Sensors Energy Storage	[228]

## Data Availability

Not applicable.
